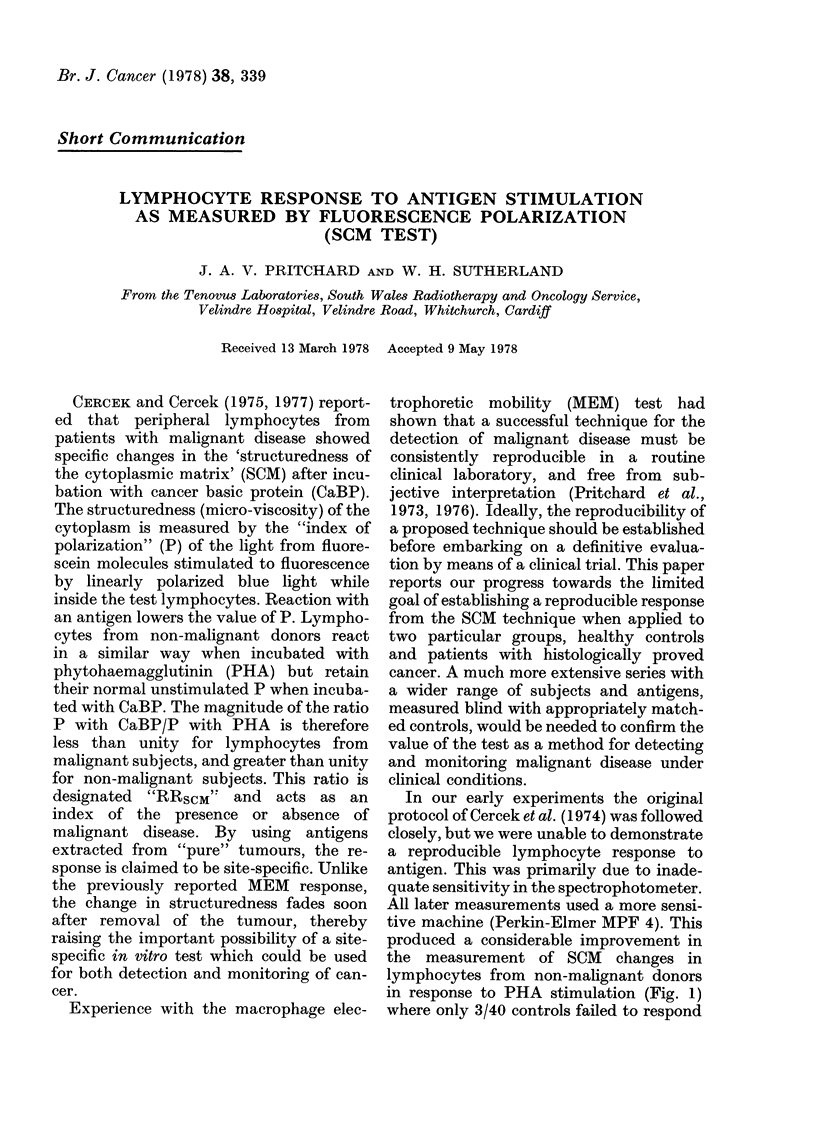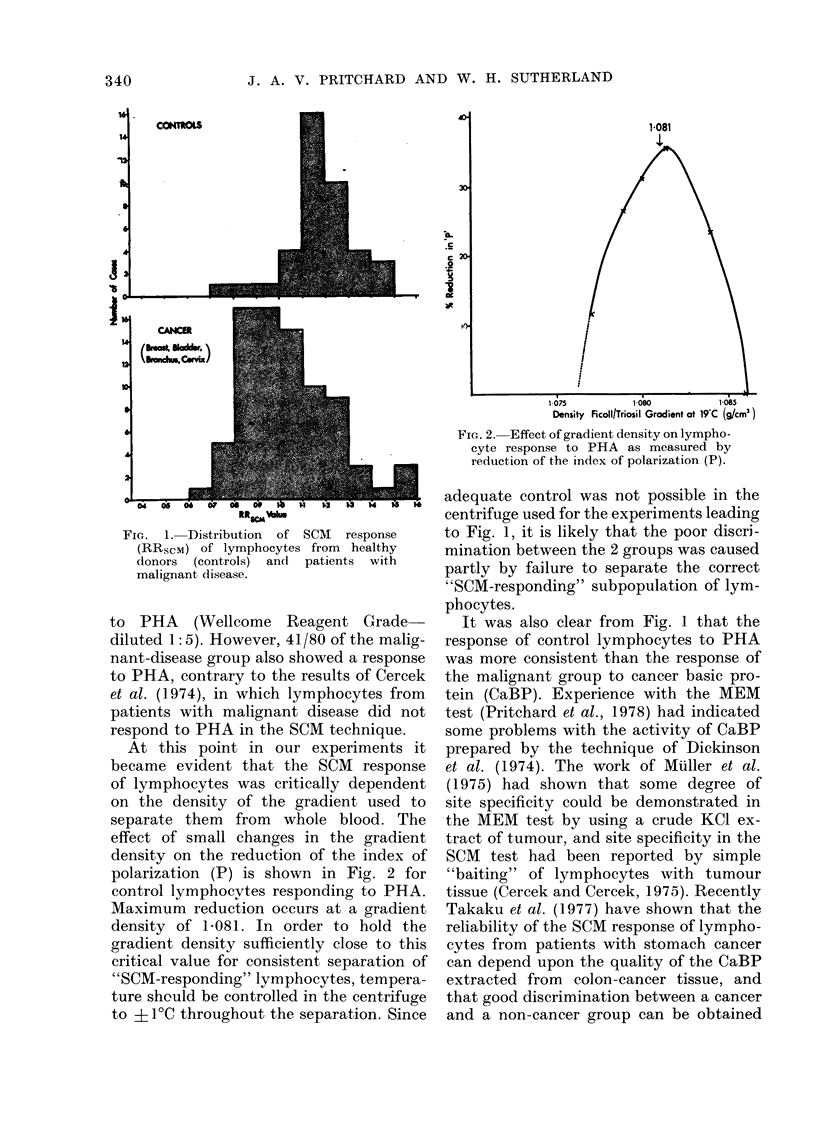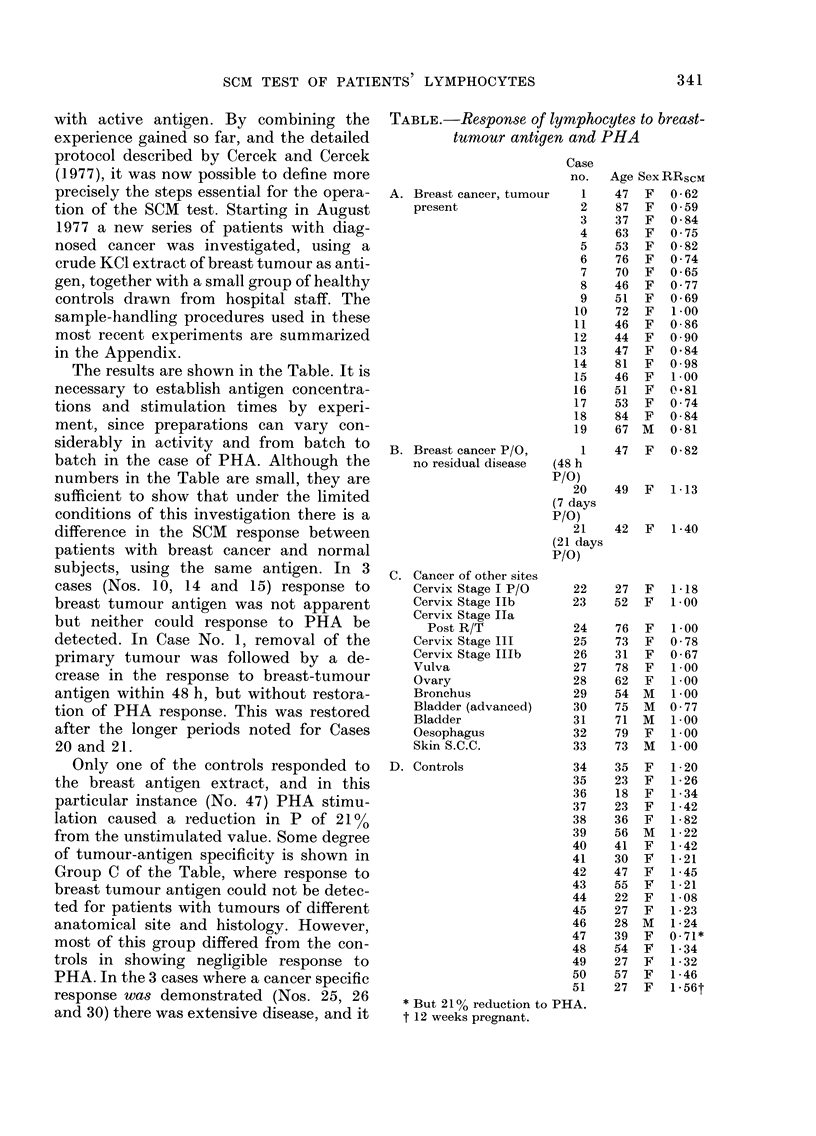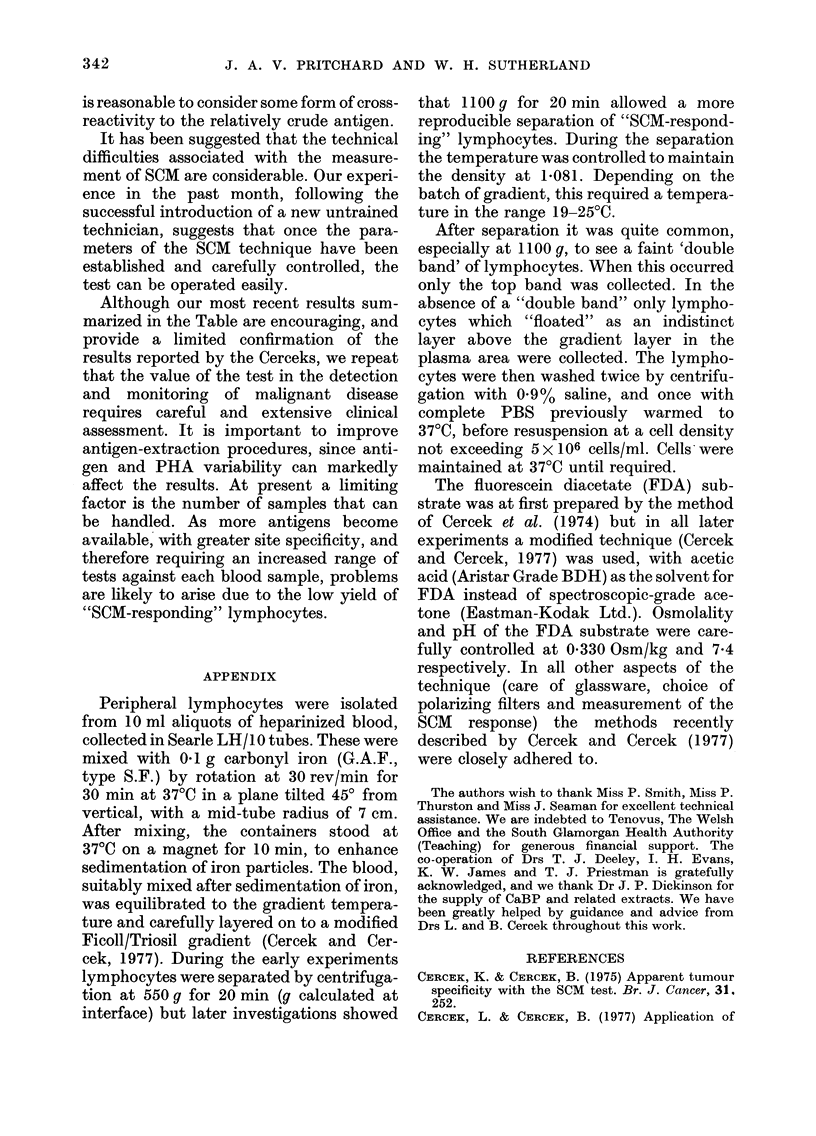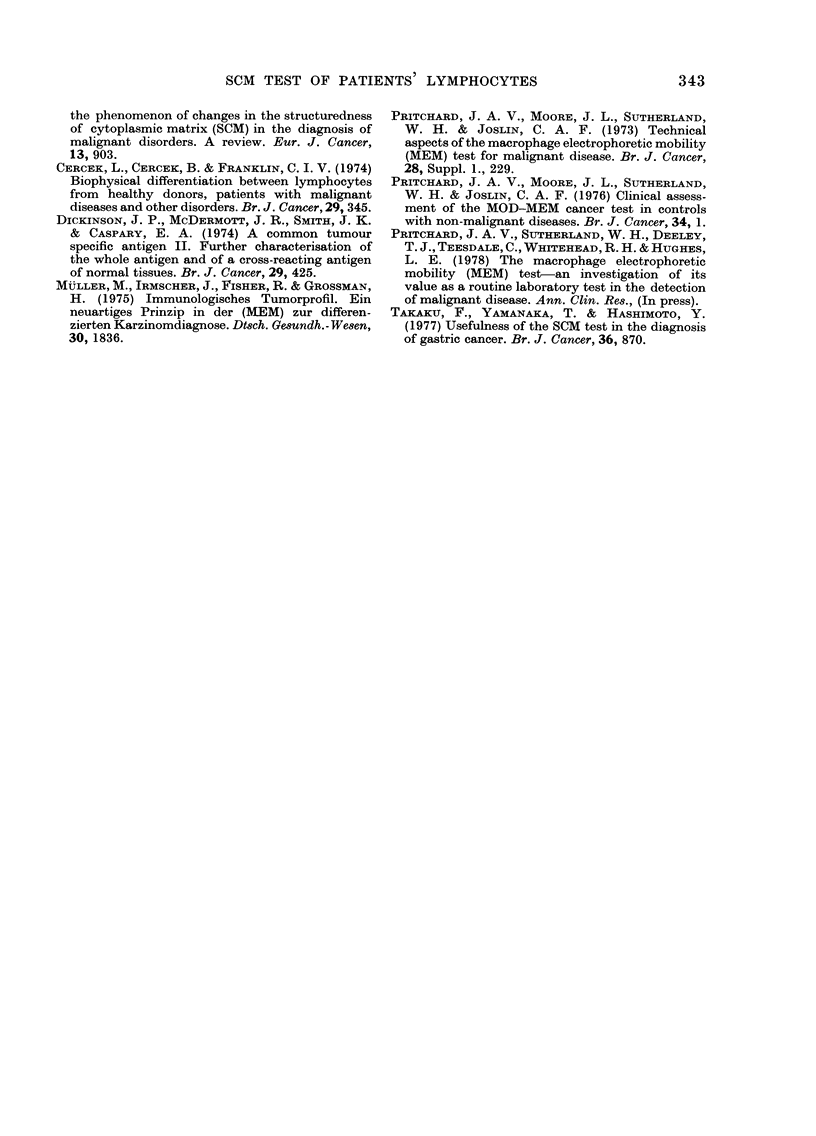# Lymphocyte response to antigen stimulation as measured by fluorescence polarization (SCM test).

**DOI:** 10.1038/bjc.1978.208

**Published:** 1978-08

**Authors:** J. A. Pritchard, W. H. Sutherland


Br. J. Cancer (1978) **38**, 339

***Short Communication***

### LYMPHOCYTE RESPONSE TO ANTIGEN STIMULATION AS MEASURED BY FLUORESCENCE POLARIZATION (SCM TEST)

J. A. V. PRITCHARD AND W. H. SUTHERLAND

*From the Tenovus Laboratories, South Wales Radiotherapy and Oncology Service, Velindre Hospital, Velindre Road, Whitchurch, Cardiff*

Received 13 March 1978 Accepted 9 May 1978

CERCEK and Cercek (1975, 1977) reported that peripheral lymphocytes from patients with malignant disease showed specific changes in the 'structuredness of the cytoplasmic matrix' (SCM) after incubation with cancer basic protein (CaBP). The structuredness (micro-viscosity) of the cytoplasm is measured by the "index of polarization" (P) of the light from fluorescein molecules stimulated to fluorescence by linearly polarized blue light while inside the test lymphocytes. Reaction with an antigen lowers the value of P. Lymphocytes from non-malignant donors react in a similar way when incubated with phytohaemagglutinin (PHA) but retain their normal unstimulated P when incubated with CaBP. The magnitude of the ratio P with CaBP/P with PHA is therefore less than unity for lymphocytes from malignant subjects, and greater than unity for non-malignant subjects. This ratio is designated "$RR_{SCM}$" and acts as an index of the presence or absence of malignant disease. By using antigens extracted from "pure" tumours, the response is claimed to be site-specific. Unlike the previously reported MEM response, the change in structuredness fades soon after removal of the tumour, thereby raising the important possibility of a site-specific *in vitro* test which could be used for both detection and monitoring of cancer.

Experience with the macrophage electrophoretic mobility (MEM) test had shown that a successful technique for the detection of malignant disease must be consistently reproducible in a routine clinical laboratory, and free from subjective interpretation (Pritchard *et al.*, 1973, 1976). Ideally, the reproducibility of a proposed technique should be established before embarking on a definitive evaluation by means of a clinical trial. This paper reports our progress towards the limited goal of establishing a reproducible response from the SCM technique when applied to two particular groups, healthy controls and patients with histologically proved cancer. A much more extensive series with a wider range of subjects and antigens, measured blind with appropriately matched controls, would be needed to confirm the value of the test as a method for detecting and monitoring malignant disease under clinical conditions.

In our early experiments the original protocol of Cercek *et al.* (1974) was followed closely, but we were unable to demonstrate a reproducible lymphocyte response to antigen. This was primarily due to inadequate sensitivity in the spectrophotometer. All later measurements used a more sensitive machine (Perkin-Elmer MPF 4). This produced a considerable improvement in the measurement of SCM changes in lymphocytes from non-malignant donors in response to PHA stimulation (Fig. 1) where only 3/40 controls failed to respond

FIG. 1.—Distribution of SCM response ($RR_{SCM}$) of lymphocytes from healthy donors (controls) and patients with malignant disease.

FIG. 2.—Effect of gradient density on lymphocyte response to PHA as measured by reduction of the index of polarization (P).

to PHA (Wellcome Reagent Grade—diluted 1:5). However, 41/80 of the malignant-disease group also showed a response to PHA, contrary to the results of Cercek *et al.* (1974), in which lymphocytes from patients with malignant disease did not respond to PHA in the SCM technique.

At this point in our experiments it became evident that the SCM response of lymphocytes was critically dependent on the density of the gradient used to separate them from whole blood. The effect of small changes in the gradient density on the reduction of the index of polarization (P) is shown in Fig. 2 for control lymphocytes responding to PHA. Maximum reduction occurs at a gradient density of 1·081. In order to hold the gradient density sufficiently close to this critical value for consistent separation of "SCM-responding" lymphocytes, temperature should be controlled in the centrifuge to $\pm 1°C$ throughout the separation. Since adequate control was not possible in the centrifuge used for the experiments leading to Fig. 1, it is likely that the poor discrimination between the 2 groups was caused partly by failure to separate the correct "SCM-responding" subpopulation of lymphocytes.

It was also clear from Fig. 1 that the response of control lymphocytes to PHA was more consistent than the response of the malignant group to cancer basic protein (CaBP). Experience with the MEM test (Pritchard *et al.*, 1978) had indicated some problems with the activity of CaBP prepared by the technique of Dickinson *et al.* (1974). The work of Müller *et al.* (1975) had shown that some degree of site specificity could be demonstrated in the MEM test by using a crude KCl extract of tumour, and site specificity in the SCM test had been reported by simple "baiting" of lymphocytes with tumour tissue (Cercek and Cercek, 1975). Recently Takaku *et al.* (1977) have shown that the reliability of the SCM response of lymphocytes from patients with stomach cancer can depend upon the quality of the CaBP extracted from colon-cancer tissue, and that good discrimination between a cancer and a non-cancer group can be obtained with active antigen. By combining the experience gained so far, and the detailed protocol described by Cercek and Cercek (1977), it was now possible to define more precisely the steps essential for the operation of the SCM test. Starting in August 1977 a new series of patients with diagnosed cancer was investigated, using a crude KCl extract of breast tumour as antigen, together with a small group of healthy controls drawn from hospital staff. The sample-handling procedures used in these most recent experiments are summarized in the Appendix.

The results are shown in the Table. It is necessary to establish antigen concentrations and stimulation times by experiment, since preparations can vary considerably in activity and from batch to batch in the case of PHA. Although the numbers in the Table are small, they are sufficient to show that under the limited conditions of this investigation there is a difference in the SCM response between patients with breast cancer and normal subjects, using the same antigen. In 3 cases (Nos. 10, 14 and 15) response to breast tumour antigen was not apparent but neither could response to PHA be detected. In Case No. 1, removal of the primary tumour was followed by a decrease in the response to breast-tumour antigen within 48 h, but without restoration of PHA response. This was restored after the longer periods noted for Cases 20 and 21.

Only one of the controls responded to the breast antigen extract, and in this particular instance (No. 47) PHA stimulation caused a reduction in P of 21% from the unstimulated value. Some degree of tumour-antigen specificity is shown in Group C of the Table, where response to breast tumour antigen could not be detected for patients with tumours of different anatomical site and histology. However, most of this group differed from the controls in showing negligible response to PHA. In the 3 cases where a cancer specific response *was* demonstrated (Nos. 25, 26 and 30) there was extensive disease, and it

TABLE.—*Response of lymphocytes to breast-tumour antigen and PHA*

| | Case no. | Age | Sex | $RR_{SCM}$ |
|---|---|---|---|---|
| A. Breast cancer, tumour present | 1 | 47 | F | 0·62 |
| | 2 | 87 | F | 0·59 |
| | 3 | 37 | F | 0·84 |
| | 4 | 63 | F | 0·75 |
| | 5 | 53 | F | 0·82 |
| | 6 | 76 | F | 0·74 |
| | 7 | 70 | F | 0·65 |
| | 8 | 46 | F | 0·77 |
| | 9 | 51 | F | 0·69 |
| | 10 | 72 | F | 1·00 |
| | 11 | 46 | F | 0·86 |
| | 12 | 44 | F | 0·90 |
| | 13 | 47 | F | 0·84 |
| | 14 | 81 | F | 0·98 |
| | 15 | 46 | F | 1·00 |
| | 16 | 51 | F | 0·81 |
| | 17 | 53 | F | 0·74 |
| | 18 | 84 | F | 0·84 |
| | 19 | 67 | M | 0·81 |
| B. Breast cancer P/O, no residual disease | 1 (48 h P/O) | 47 | F | 0·82 |
| | 20 (7 days P/O) | 49 | F | 1·13 |
| | 21 (21 days P/O) | 42 | F | 1·40 |
| C. Cancer of other sites | | | | |
| Cervix Stage I P/O | 22 | 27 | F | 1·18 |
| Cervix Stage IIb | 23 | 52 | F | 1·00 |
| Cervix Stage IIa Post R/T | 24 | 76 | F | 1·00 |
| Cervix Stage III | 25 | 73 | F | 0·78 |
| Cervix Stage IIIb | 26 | 31 | F | 0·67 |
| Vulva | 27 | 78 | F | 1·00 |
| Ovary | 28 | 62 | F | 1·00 |
| Bronchus | 29 | 54 | M | 1·00 |
| Bladder (advanced) | 30 | 75 | M | 0·77 |
| Bladder | 31 | 71 | M | 1·00 |
| Oesophagus | 32 | 79 | F | 1·00 |
| Skin S.C.C. | 33 | 73 | M | 1·00 |
| D. Controls | 34 | 35 | F | 1·20 |
| | 35 | 23 | F | 1·26 |
| | 36 | 18 | F | 1·34 |
| | 37 | 23 | F | 1·42 |
| | 38 | 36 | F | 1·82 |
| | 39 | 56 | M | 1·22 |
| | 40 | 41 | F | 1·42 |
| | 41 | 30 | F | 1·21 |
| | 42 | 47 | F | 1·45 |
| | 43 | 55 | F | 1·21 |
| | 44 | 22 | F | 1·08 |
| | 45 | 27 | F | 1·23 |
| | 46 | 28 | M | 1·24 |
| | 47 | 39 | F | 0·71* |
| | 48 | 54 | F | 1·34 |
| | 49 | 27 | F | 1·32 |
| | 50 | 57 | F | 1·46 |
| | 51 | 27 | F | 1·56† |

* But 21% reduction to PHA.

† 12 weeks pregnant.

is reasonable to consider some form of cross-reactivity to the relatively crude antigen.

It has been suggested that the technical difficulties associated with the measurement of SCM are considerable. Our experience in the past month, following the successful introduction of a new untrained technician, suggests that once the parameters of the SCM technique have been established and carefully controlled, the test can be operated easily.

Although our most recent results summarized in the Table are encouraging, and provide a limited confirmation of the results reported by the Cerceks, we repeat that the value of the test in the detection and monitoring of malignant disease requires careful and extensive clinical assessment. It is important to improve antigen-extraction procedures, since antigen and PHA variability can markedly affect the results. At present a limiting factor is the number of samples that can be handled. As more antigens become available, with greater site specificity, and therefore requiring an increased range of tests against each blood sample, problems are likely to arise due to the low yield of "SCM-responding" lymphocytes.

### APPENDIX

Peripheral lymphocytes were isolated from 10 ml aliquots of heparinized blood, collected in Searle LH/10 tubes. These were mixed with 0·1 g carbonyl iron (G.A.F., type S.F.) by rotation at 30 rev/min for 30 min at 37°C in a plane tilted 45° from vertical, with a mid-tube radius of 7 cm. After mixing, the containers stood at 37°C on a magnet for 10 min, to enhance sedimentation of iron particles. The blood, suitably mixed after sedimentation of iron, was equilibrated to the gradient temperature and carefully layered on to a modified Ficoll/Triosil gradient (Cercek and Cercek, 1977). During the early experiments lymphocytes were separated by centrifugation at 550 *g* for 20 min (*g* calculated at interface) but later investigations showed that 1100 *g* for 20 min allowed a more reproducible separation of "SCM-responding" lymphocytes. During the separation the temperature was controlled to maintain the density at 1·081. Depending on the batch of gradient, this required a temperature in the range 19–25°C.

After separation it was quite common, especially at 1100 *g*, to see a faint 'double band' of lymphocytes. When this occurred only the top band was collected. In the absence of a "double band" only lymphocytes which "floated" as an indistinct layer above the gradient layer in the plasma area were collected. The lymphocytes were then washed twice by centrifugation with 0·9% saline, and once with complete PBS previously warmed to 37°C, before resuspension at a cell density not exceeding $5 \times 10^6$ cells/ml. Cells were maintained at 37°C until required.

The fluorescein diacetate (FDA) substrate was at first prepared by the method of Cercek *et al.* (1974) but in all later experiments a modified technique (Cercek and Cercek, 1977) was used, with acetic acid (Aristar Grade BDH) as the solvent for FDA instead of spectroscopic-grade acetone (Eastman-Kodak Ltd.). Osmolality and pH of the FDA substrate were carefully controlled at 0·330 Osm/kg and 7·4 respectively. In all other aspects of the technique (care of glassware, choice of polarizing filters and measurement of the SCM response) the methods recently described by Cercek and Cercek (1977) were closely adhered to.

The authors wish to thank Miss P. Smith, Miss P. Thurston and Miss J. Seaman for excellent technical assistance. We are indebted to Tenovus, The Welsh Office and the South Glamorgan Health Authority (Teaching) for generous financial support. The co-operation of Drs T. J. Deeley, I. H. Evans, K. W. James and T. J. Priestman is gratefully acknowledged, and we thank Dr J. P. Dickinson for the supply of CaBP and related extracts. We have been greatly helped by guidance and advice from Drs L. and B. Cercek throughout this work.

### REFERENCES

CERCEK, K. & CERCEK, B. (1975) Apparent tumour specificity with the SCM test. *Br. J. Cancer*, **31**, 252.

CERCEK, L. & CERCEK, B. (1977) Application of the phenomenon of changes in the structuredness of cytoplasmic matrix (SCM) in the diagnosis of malignant disorders. A review. *Eur. J. Cancer*, **13**, 903.

CERCEK, L., CERCEK, B. & FRANKLIN, C. I. V. (1974) Biophysical differentiation between lymphocytes from healthy donors, patients with malignant diseases and other disorders. *Br. J. Cancer*, **29**, 345.

DICKINSON, J. P., MCDERMOTT, J. R., SMITH, J. K. & CASPARY, E. A. (1974) A common tumour specific antigen II. Further characterisation of the whole antigen and of a cross-reacting antigen of normal tissues. *Br. J. Cancer*, **29**, 425.

MÜLLER, M., IRMSCHER, J., FISHER, R. & GROSSMAN, H. (1975) Immunologisches Tumorprofil. Ein neuartiges Prinzip in der (MEM) zur differenzierten Karzinomdiagnose. *Dtsch. Gesundh.-Wesen*, **30**, 1836.

PRITCHARD, J. A. V., MOORE, J. L., SUTHERLAND, W. H. & JOSLIN, C. A. F. (1973) Technical aspects of the macrophage electrophoretic mobility (MEM) test for malignant disease. *Br. J. Cancer*, **28**, Suppl. 1., 229.

PRITCHARD, J. A. V., MOORE, J. L., SUTHERLAND, W. H. & JOSLIN, C. A. F. (1976) Clinical assessment of the MOD–MEM cancer test in controls with non-malignant diseases. *Br. J. Cancer*, **34**, 1.

PRITCHARD, J. A. V., SUTHERLAND, W. H., DEELEY, T. J., TEESDALE, C., WHITEHEAD, R. H. & HUGHES, L. E. (1978) The macrophage electrophoretic mobility (MEM) test—an investigation of its value as a routine laboratory test in the detection of malignant disease. *Ann. Clin. Res.*, (In press).

TAKAKU, F., YAMANAKA, T. & HASHIMOTO, Y. (1977) Usefulness of the SCM test in the diagnosis of gastric cancer. *Br. J. Cancer*, **36**, 870.